# Optoelectronic Properties of Van Der Waals Hybrid Structures: Fullerenes on Graphene Nanoribbons

**DOI:** 10.3390/nano7030069

**Published:** 2017-03-20

**Authors:** Julián David Correa, Pedro Alejandro Orellana, Mónica Pacheco

**Affiliations:** 1 Departamento de Ciencias Básicas, Universidad de Medellín, 050026 Medellín, Colombia; jcorrea@udem.edu.co; 2 Departamento de Física, Universidad Técnica Federico Santa María, 2340000 Valparaíso, Chile; pedro.orellana@usm.cl

**Keywords:** graphene, fullerene, nanoribons

## Abstract

The search for new optical materials capable of absorbing light in the frequency range from visible to near infrared is of great importance for applications in optoelectronic devices. In this paper, we report a theoretical study of the electronic and optical properties of hybrid structures composed of fullerenes adsorbed on graphene and on graphene nanoribbons. The calculations are performed in the framework of the density functional theory including the van der Waals dispersive interactions. We found that the adsorption of the C${}_{60}$ fullerenes on a graphene layer does not modify its low energy states, but it has strong consequences for its optical spectrum, introducing new absorption peaks in the visible energy region. The optical absorption of fullerenes and graphene nanoribbon composites shows a strong dependence on photon polarization and geometrical characteristics of the hybrid systems, covering a broad range of energies. We show that an external electric field across the nanoribbon edges can be used to tune different optical transitions coming from nanoribbon–fullerene hybridized states, which yields a very rich electro-absorption spectrum for longitudinally polarized photons. We have carried out a qualitative analysis on the potential of these hybrids as possible donor-acceptor systems in photovoltaic cells.

## 1. Introduction

Nanostructures based on carbon allotropes have been intensively studied in the last two decades due to the possibility of modulating and controlling their optical and transport properties for novel optoelectronic applications [1] and development of photovoltaic devices [2,3]. Graphene-based nanostructures are highlighted for their versatility and excellent optical, electronic and transport properties, which can be manipulated by changing the geometry of the structure, applying external fields or by the incorporation of impurities and functionalization with foreign substances such as organic molecules [4,5].

Several hybrid nanostructures with particular geometric configurations are formed with different carbon allotropes through covalent or non-covalent interactions, with complexes such as graphene–nanotubes, graphene–fullerenes, nanoribbon–fullerenes, nanotube–fullerenes and nanobuds [6]. These have been proposed and synthesized for many applications in optoelectronics, photonics, energy storage and solar cells. The most studied system has been the C${}_{60}$–graphene composite, in particular for applications in lithium batteries, electrodes for photovoltaics applications, supercapacitors, [7] and it has been studied in other fields such as organic thermoelectric materials [8]. Very recently, vertical heterostructures composed of C${}_{60}$ thin film on graphene have been assembled, and vertical graphene transistors were able to be fabricated [9].

It is known by experimental and theoretical studies that the adsorption of fullerenes on pristine graphene is governed by van der Waals interactions [10,11]. Computational studies on non-covalent interactions of carbon and boron fullerenes with graphene, performed by A. K. Manna and S. K. Pati [12], have shown that fullerenes physisorbed on graphene monolayers are stable and have charge transfer between the components of the complex. The above behavior is a necessary condition to ensure the photocurrent in photovoltaic devices.

In this paper, we study theoretically the electronic structure and optical and electro-optical properties of graphene–fullerene nanohybrids. In the framework of the density functional theory (DFT), we have calculated the electronic band structure, the density of states (DOS) and the optical spectrum of two different types of hybridized configurations: C${}_{60}$ fullerenes physisorbed on a graphene monolayer and graphene nanoribbons functionalized with different fullerene molecules C${}_{20}$, C${}_{60}$, C${}_{70}$ and the derivative [6,6]-phenyl-C61-butyric acid methyl ester (PCMB).

Our results show that the energy spectrum and DOS for all hybrid structures are an ensemble of the overall features of its components, which is an expected result due to the type of interactions between the components. We found that the adsorption of the C${}_{60}$ fullerenes on the single layer graphene (SLG) does not modify the low energy states of the pristine graphene maintaining its metallic character, but it has strong consequences on its optical spectrum, introducing two new absorption peaks in the visible energy range. The interaction between both fullerenes enriches even more the band structure and absorption spectra in the SLG.

It is well known that graphene nanoribbons hold unique geometrical-dependent electronical properties that can be tuned by varying the width of the nanoribbon and the structure of its edges [13]. Our calculations show that complexes formed by fullerenes adsorbed on armchair graphene nanoribbons (GR) display very interesting electronic and optical properties due to the interplay effects between the ribbon finite widths and the fullerene size. The presence of the adsorbed fullerenes induces the appearance of extra peaks in the GR optical spectra. The number and energy position of the peaks are strongly dependent on the type of adsorbed fullerene and on the ribbon width. We show that, in these complexes, the linear absorption spectra for longitudinally and transversely polarized photons cover a broad energy region of the optical spectrum.

In addition, we included an external electric field applied across the ribbon edges and we investigated the electro-absorption spectrum of two representative hybrids GR–C${}_{60}$ and GR–C${}_{70}$. We found that some original absorption peaks associated with the pristine GR selection rules are suppressed while new extra peaks appear in the optical spectra.

## 2. Results and Discussion

In Table 1, we show some geometrical and electronic structure parameters for each complex studied. Three different widths of GRs are considered: 10.635 Å (8GR), 13.144 Å (10GR) and 15.629 Å (12GR). When the fullerenes are physisorbed on SLG or nGRs (n = 8, 10 or 12), the equilibrium distance of the fullerenes varies between d=2.060 Å and d=3.001 Å. These distances depend on several factors such as the contact area, the widths of the nGRs, the size, shape and orientation of the fullerenes, and the vdW exchange-correlation functional employed. Our results of the binding energy of fullerenes on SLG and nGR are in the range of 0.44 to 0.76 eV, where the largest value corresponds to the 10GR–C${}_{70}$ system, and the smallest value to the 8GR–C${}_{20}$ system. The difference between the smallest and the largest value of the binding energy is due to the decrease in the contact area. This is confirmed by the results displayed in Table 1, where we observe that the largest binding energy, for each nGR–fullerene system, corresponds to the C${}_{70}$ fullerene, which is the biggest fullerene considered in this work.

The adsorption energy is also influenced by the finite width of the nGRs, and in Table 1 it is possible to compare the values of the $E_{b}$ corresponding to the different systems. In all cases, we observe that the binding energy for nGR–C${}_{60}$ is lower than that corresponding to the SLG–C${}_{60}$ system, which suggests that the edges of the nGRs induce a reduction in the binding of the fullerenes. Our result for the binding energy of SLG–C${}_{60}$ is 0.74 eV, and this value is in agreement with previous theoretical calculations [14], that inform on a value of $E_{b}=0.85$ eV using a vdW-DF1 level of theory. This last result is consistent with the reported experimental value for C${}_{60}$ physisorbed on graphite [15]. In contrast, the adsorption energy of all the fullerenes increases with the width of the GRs because the edges effect becomes less important.

Finally, in Table 1, the band gap associated with the nGR ($E_{g}$) and the difference in energy between the high occupied molecular orbital (HOMO) and the low occupied molecular orbital (LUMO) associated with the fullerenes is displayed. The results show that this difference in energy is almost invariant for each complex, which suggests that the physical properties of the pristine SLG and nGRs, and those of the bare fullerenes, are preserved in the complex.

### 2.1. Fullerenes Adsorbed on a Graphene Monolayer

**Figure 1:**
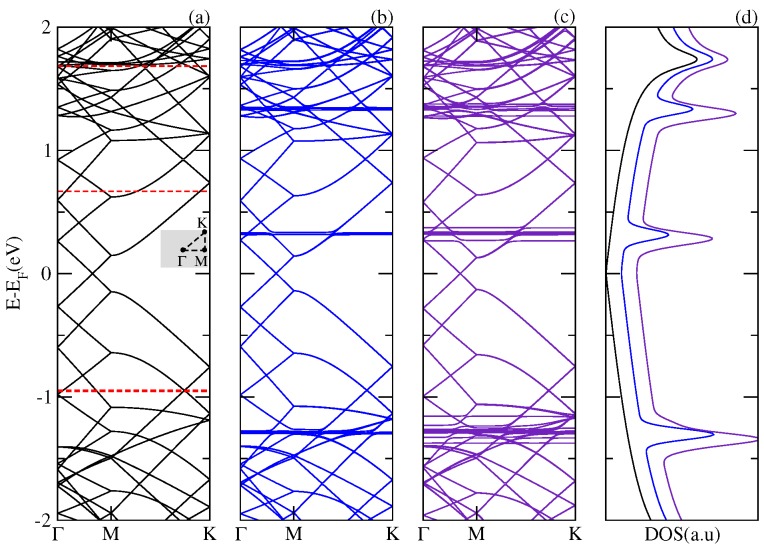
Electronic band structure of (**a**) a supercell of a graphene monolayer; the red line dash represents the energy levels of isolated C${}_{60}$; (**b**) a C${}_{60}$ fullerene physisorbed onto the supercell of a graphene monolayer and (**c**) two C${}_{60}$ fullerenes physisorbed onto the supercell of a graphene monolayer; Panel (**d**) shows the total density of states.

Figure 1 shows the electronic band structure and the total DOS of the single layer graphene and of the complexes composed of a C${}_{60}$ physisorbed on a graphene layer (SLG–C${}_{60}$) and two fullerenes adsorbed on a graphene layer (SLG–2C${}_{60}$). It is apparent from the low energies results that the energy spectrum of the hybrid structure SLG–C${}_{60}$ is basically a superposition of the energy spectrum corresponding to each component of the composite. The energy levels of the C${}_{60}$ molecule appear as dispersionless bands around the Fermi energy (E${}_{F}$) of the system. The presence of these states is reflected in the DOS as resonances inserted between the characteristic Van Hove peaks of the graphene. The same overall features are observed for the SLG–2C${}_{60}$ complex; however, in this case, the interaction between the two fullerenes splits the levels forming stripes of dispersionless bands around the original levels of the fullerene. The resonances in the DOS are enhanced and slightly displaced with respect to those corresponding to the SLG–C${}_{60}$ system. We found that the adsorption of the C${}_{60}$ fullerenes on the SLG does not modify the low energy states of the pristine graphene maintaining its metallic character.

**Figure 2:**
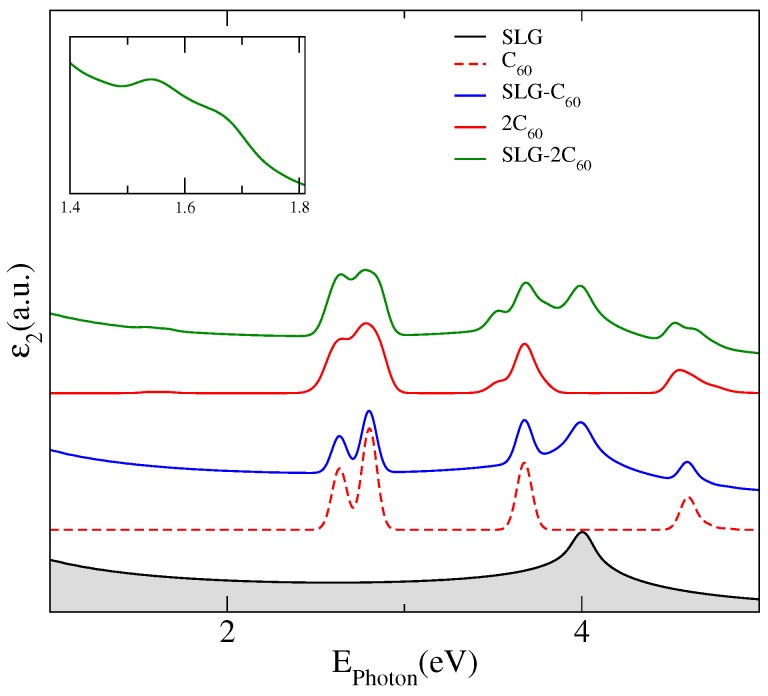
Imaginary part of the dielectric constant as a function of the photon energy for a pristine single layer graphene ( black line), isolated C${}_{60}$ (red dashed line) and 2C${}_{60}$ (red line) fullerenes and, for the SLG–C${}_{60}$ (blue line) and SLG–2C${}_{60}$ (green line) hybrids. The insert shows a zoom of the imaginary part of the dielectric constant of SLG–2C${}_{60}$.

The main effects of the adsorption of fullerenes on graphene are manifested in its optical response, which is displayed in Figure 2 where we have plotted the imaginary part of the dielectric constant for a pristine SLG (black line), an isolated C${}_{60}$ (dashed red line) and 2C${}_{60}$ fullerenes (red line) and, for the complexes SLG–C${}_{60}$ (blue line) and SLG–2C${}_{60}$ (green line). The absorption spectra of the hybrid systems present noticeable differences with the SLG spectrum in the visible energy region where two prominent peaks appear from interband transitions between the new states stemming from the adsorbed fullerenes. The extra interband transitions, at the high energy region, also modify the distinctive absorption peak in graphene coming from band-to-band transitions near the saddle-point singularity at the M-point. Unlike the case of one C${}_{60}$ adsorbed on a SLG, for which the optical spectrum of the hybrid system is mainly a superposition of the spectrum of each component, in the complex SLG–2C${}_{60}$ the interaction between both fullerenes produces a symmetry breaking leading to a splitting of the peaks associated to transitions between fullerene levels, which can be observed at energies of about 3.5 eV and at 4.5 eV in the corresponding spectrum. However, the most remarkable result for the optical spectrum of the hybrid SLG–2C${}_{60}$ is the presence of a low-energy absorption peak located at 1.54 eV, which is not attributable to any transition between graphene energy levels or between fullerene levels but seems to correspond to transitions between graphene–fullerene strongly hybridized states to levels of the first flat miniband associated to C${}_{60}$ levels that are forbidden for isolated fullerenes on graphene. We have included an inset in Figure 2 to highlight this peak, which should be more intense for a greater number of interacting molecules.

### 2.2. Fullerenes Adsorbed on Armchair Nanoribbons

**Figure 3:**
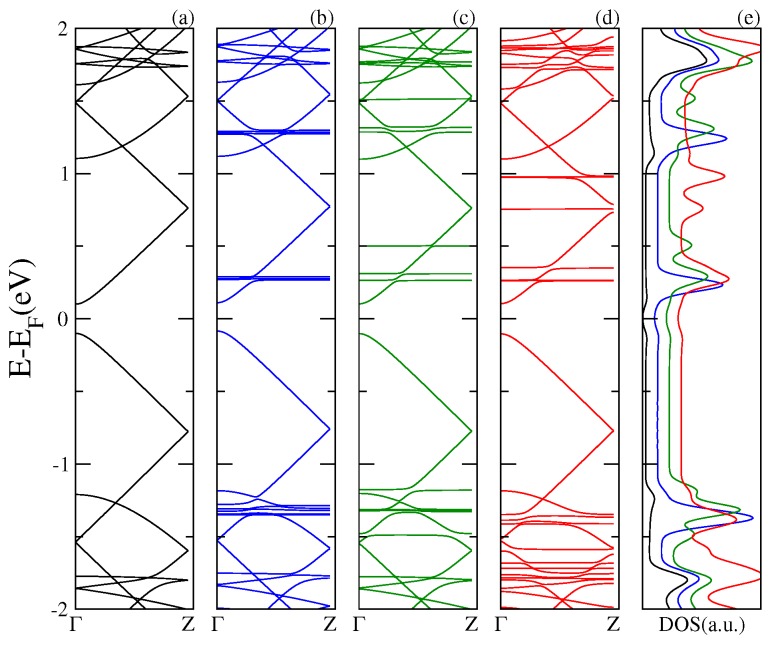
Electronic band structure of the 8GR–fullerene system for a wave vector along the *z* direction. Each panel displays the energy bands for the pristine 8GR in (**a**), and for the different hybrid systems: 8GR–C${}_{60}$ in (**b**), 8GR–PCMB in (**c**), and 8GR–C${}_{70}$ in (**d**). Panel (**e**) shows the total DOS.

**Figure 4:**
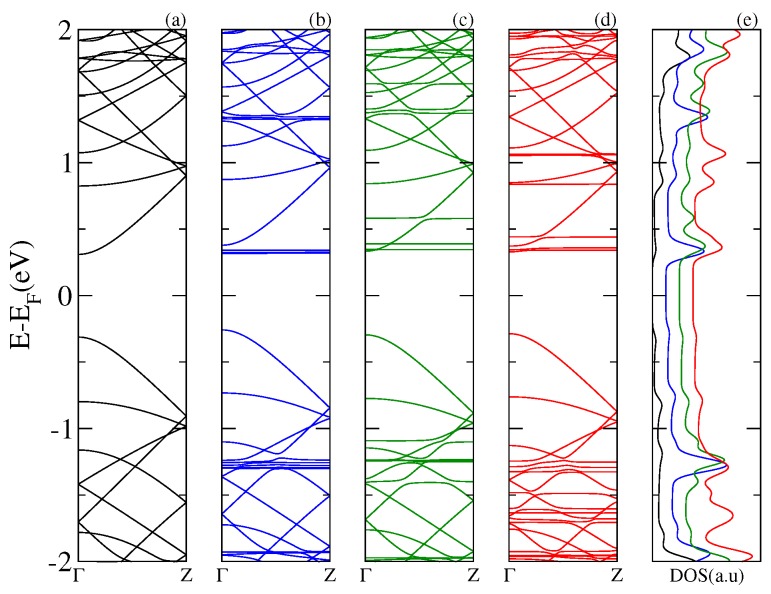
Electronic band structure of the 12GR–fullerene system for a wave vector along the *z* direction. Each panel displays the energy bands for the pristine 12GR in (**a**), and for the different hybrid systems: 12GR–C${}_{60}$ in (**b**), 12GR–PCMB in (**c**), and 12GR–C${}_{70}$ in (**d**). Panel (**e**) shows the total DOS.

**Figure 5:**
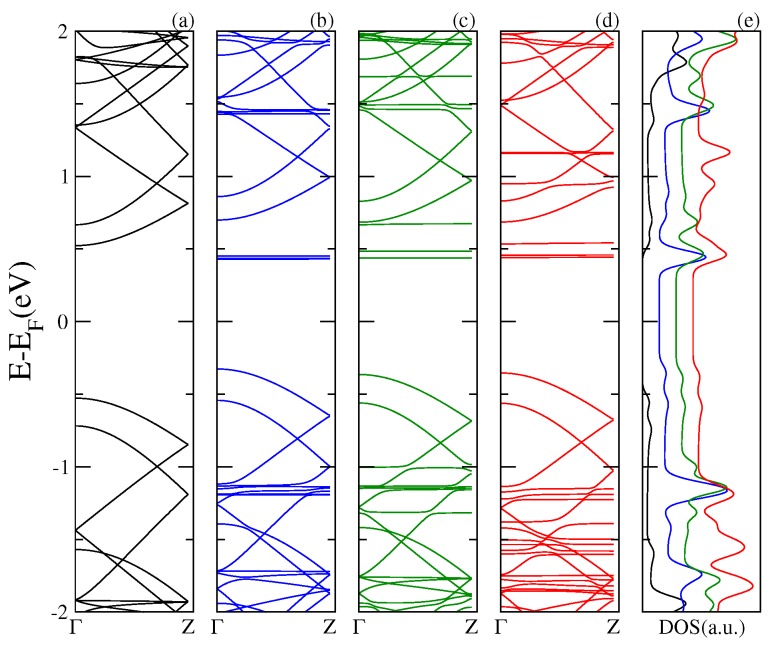
Electronic band structure of the 10GR–fullerene system for a wave vector along the *z* direction. Each panel displays the energy bands for the pristine 10GR in (**a**), and for the different hybrid systems: 10GR–C${}_{60}$ in (**b**), 10GR–PCMB in (**c**), and 10GR–C${}_{70}$ in (**d**). Panel (**e**) shows the total DOS.

Next, in Figure 3, Figure 4 and Figure 5, we display the electronic band structure and corresponding DOS for armchair nanoribbons of different widths: nGR, n = 8, 12, 10. The band spectrum is plotted as a function of the wave vector along the *z* direction. Each figure shows results for the (a) pristine nGR, and the hybrids: (b) nGR–C${}_{60}$; (c) nGR–PCMB; and (d) nGR–C${}_{70}$. In (e) the corresponding DOS for the nanostructures is depicted.

Similarly, as in the case of the SLG–fullerenes systems, the energy spectrum of nGRs with adsorbed fullerenes displays a superposition of *π* bands coming from the pristine nanoribbon and localized states generated by the adsorption of the fullerene molecules. As a general observation, we can infer that the modification of the electronic structure of each nGR due to the fullerene adsorption is strongly dependent on the size of the fullerene molecule. The DOS for the studied complexes shows the emergence of many new resonances apart from those proper of the geometrical characteristics of the nanoribbons.

Our results show that the main alterations in the electronic band structure of the pristine nanoribbons occur for the GRs of smaller band gap, 8GR (Figure 3) and 12GR (Figure 4). For these cases, the *π* bands of the graphene nanoribbons are clearly modified as a consequence of the re-hybridization with the molecule orbitals. In particular, this is more evident in Figure 4 for the complexes with molecules adsorbed on the narrowest nanoribbon in which the states near the Fermi energy are strongly affected. This is manifested in the corresponding DOS as an increase of the resonance strengths.

**Figure 6:**
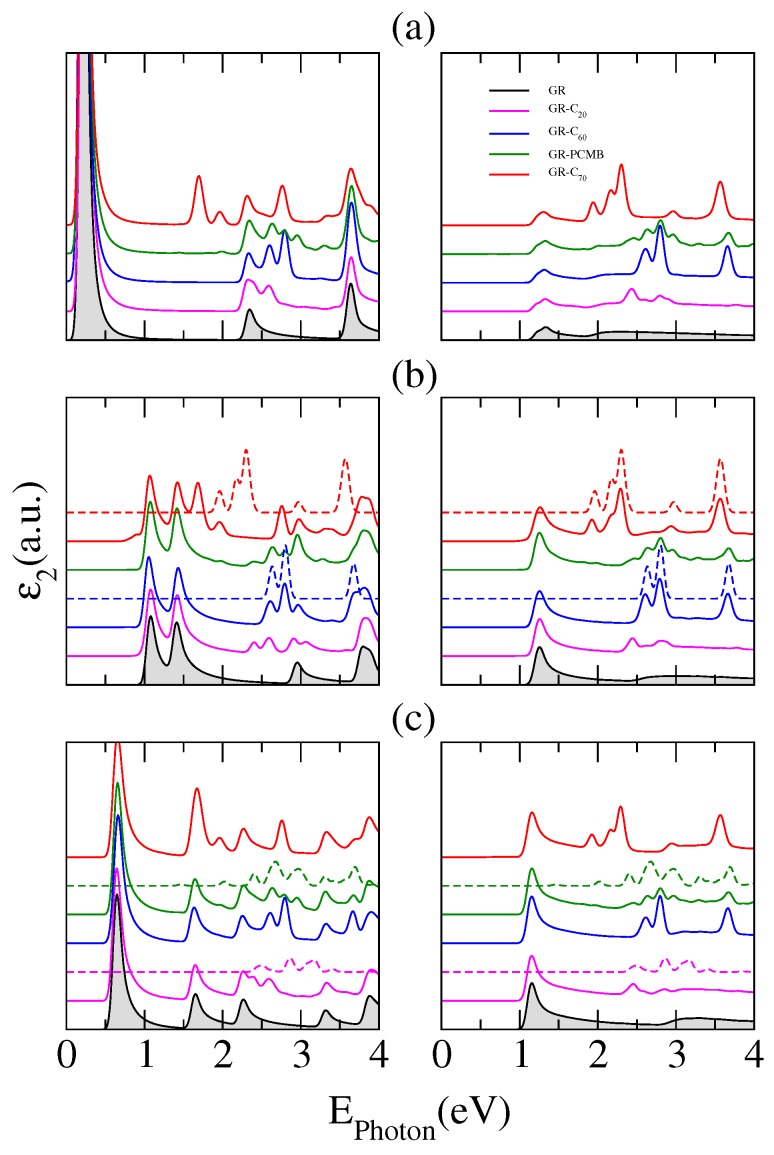
Imaginary part of the dielectric constant for fullerenes adsorbed on (**a**) 8GR; (**b**) 10GR and (**c**) 12GR nanoribbons. The curves in each panel have been vertically shifted for clarity. The spectra of the pristine nanoribbons are shadowed in gray and the spectra of bare fullerenes are displayed in dashed curves. The left panels are for parallel light polarization and the right panels for perpendicular light polarization.

The optical response of the hybrid structures shows many rich features, as noted in Figure 6 where the corresponding absorption spectra are plotted. Distinct panels present calculations of the imaginary part of the dielectric constant as a function of the photon energy for fullerene molecules adsorbed on (a) 8GR; (b) 10GR and (c) 12GR nanoribbons. The curves displayed in each panel correspond to the optical spectrum of the different complexes, vertically shifted for clarity. We have also included results for the molecule C${}_{20}$, the smallest known fullerene. For comparison, each panel incorporates—shadowed in gray—the corresponding optical response of the pristine nanoribbon forming the complex. The left panels exhibit the optical response for parallel light polarization (along the length of the ribbon-*z*) and the right panels for perpendicular light polarization (across the ribbon-*x*). It can be observed that the principal signature of the adsorption of molecules on the nanoribbons is the strong light absorption in the range between 2 and 3 eV. This is true for all nanoribbons considered, independent of their width and of the light polarization considered.

In the range of lower energies (E${}_{\mathrm{Photon}}<2$ eV) and for longitudinally polarized photons, the optical absorption of the different systems shows a strong dependence on the nanoribbon width, exhibiting a greater number of absorption peaks for wider nanoribbons. This is an expected behavior for pristine GRs [16]. In this range of energy, the optical response is dominated by the nanoribbon electronic properties, except for the nGR–C${}_{70}$ systems due to the stronger interaction between the parts of the complex. For these systems, we can realize the appearance of new transitions between states hybridized of the nanoribbon–fullerene complex.

**Figure 7:**
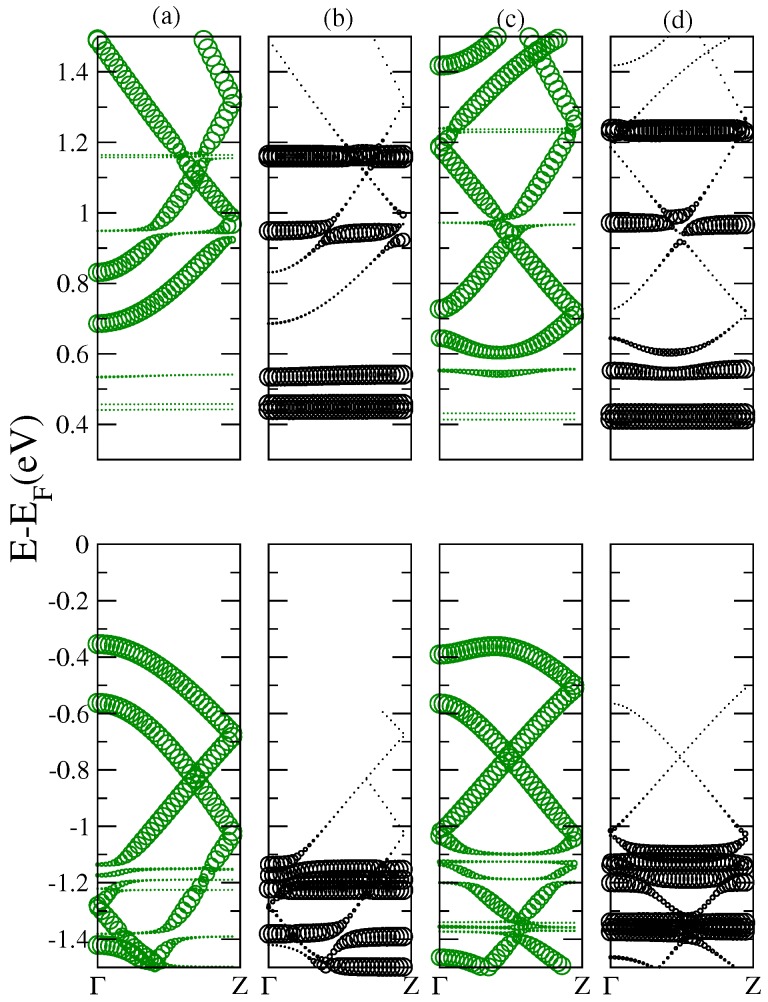
Fat-band representation for the band structure of the complex 10GR–C${}_{70}$. Columns (**a**) and (**b**) are for a null electric field and columns (**c**) and (**d**) for an electric field of 0.5 V/Å. Columns (**a**) and (**c**) show the contribution of the 10GR carbon atoms and columns (**b**) and (**d**) the contribution of the C${}_{70}$ carbon atoms to the band structure. Upper panels correspond to conduction bands and lower panels correspond to the valence bands.

For higher energy regions, the interpretation of the origin of the different absorption peaks is more complicated due to the superposition, or hybridization of the nanoribbon and fullerene states. For comparison, we have included in dashed lines the optical response of isolated C${}_{70}$ and C${}_{60}$ fullerenes in Figure 6b, and in Figure 6c those of isolated PCMB and C${}_{20}$. This effectively permits us to realize the unveiling of new optical resonances derived from transitions between hybridized states of the nanoribbons and fullerenes. The strong peak at about 1.69 eV for the complex 10GR–C${}_{70}$ is conspicuous, and it does not correspond to any transition between states associated to the C${}_{70}$ molecule or to transitions between states associated to the nanoribbon, but clearly it corresponds to a transition between hybridized states. This can be observed in the columns (a) and (b) of Figure 7 where the electronic contribution to the band structure of the complex, coming from the carbon atoms of the nanoribbon and those from the atoms of the C${}_{70}$, has been calculated separately (fat-band representation).

It has been shown before that GRs exhibit a strongly anisotropic optical response due to the symmetry selection rules [16], and this can be observed in Figure 6 for the absorption spectra of pristine nanoribbons where only one absorption peak, located at low photon energies, is noticeable for light polarized perpendicular to the nanoribbons (shadow curves). This peak remains without distortion for all considered hybrid structures and clearly corresponds to transitions between nanoribbon states. Unlike the case of polarization along the nanoribbon edges, at the high energy range the optical spectra for transversely polarized light show only resonant transitions between states of isolated fullerenes. This is clear from the comparison between the spectra of the hybrid structures and the bare fullerenes (dashed curves). Thereby, the absorption spectra for transversely polarized photons of the nGR–fullerene systems enable us to distinguish those peaks resulting from transitions between fullerene states, between nanoribbon states, or from transitions between hybridized states of the complex.

**Figure 8:**
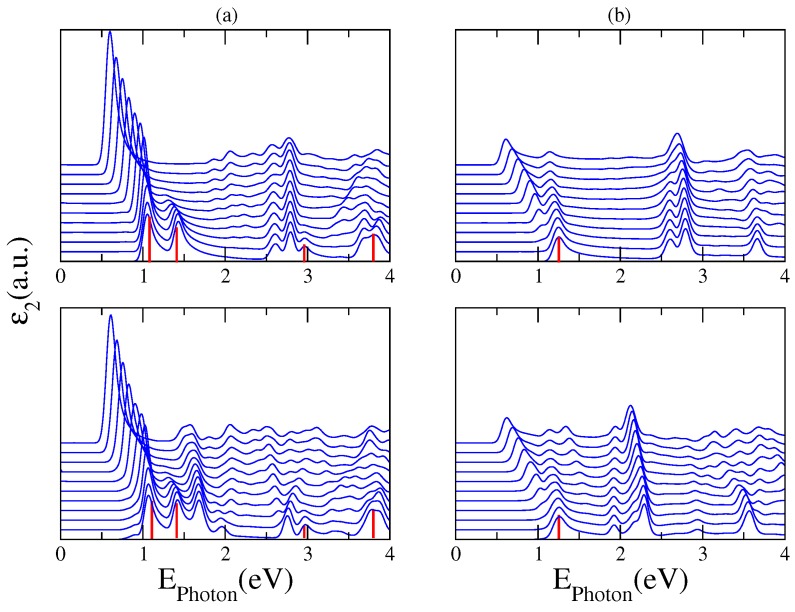
Imaginary part of the dielectric constant for a 10GR–C${}_{60}$ (upper panel) and a 10GR–C${}_{70}$ (lower panel) system for incident light linearly polarized (**a**) parallel to the ribbon and (**b**) perpendicular to the ribbon. The curves correspond to electric field intensities from 0.0 to 1.0 V/Å, vertically shifted for clarity. The red line shows the position of the optical absorption peaks of the pristine 10GR.

The optical transitions of the nanoribbon–fullerene hybrid systems can be tuned by the application of an external static electric field across the nanoribbon edges. We have calculated the electro-absorption spectrum of two representative hybrids which have shown a rich absorption spectra in the visible range of light. Figure 8 displays calculations of the imaginary part of the dielectric function for a 10GR–C${}_{60}$ (upper panel) and a 10GR–C${}_{70}$ (lower panel) system, for incident light linearly polarized (a) parallel to the ribbon and (b) perpendicular to the ribbon. The curves displayed in both panels correspond to different electric field intensities, scanned from 0.0 to 1.0 V/Å, and vertically shifted for clarity. The position of the optical absorption peaks of the pristine 10GR has been highlighted with a red line for comparison. It is clear from the results that due to the breaking of symmetry caused by the electric field, some original absorption peaks associated with the pristine GR are suppressed, while new extra peaks associated with forbidden transitions for zero electric fields, appear in the optical spectra. The common feature observed in the optical spectra of both structures is the characteristic redshift of the absorption edge induced by the electric field. This is an expected result because the structure of the infrared absorption mainly originates from transitions between states of the 10GR [13,17]. For photon energies higher than 1.5 eV, it is possible to differentiate two types of resonances in terms of its dependence with the electric field intensity. The magnitude of the absorption peaks associated with transitions between nanoribbon states increases with the field strength, and the corresponding positions move to lower energies. In contrast, the position of the resonances associated with transitions between fullerene states is not strongly affected by the electric field except for the intensities considered.

An important result provided by the electro-absorption spectra is the feasibility to analyze and detect those optical resonances derived from transitions between states of an energy-band of the nanoribbon to energy levels of the fullerene. Let us look for instance at the absorption peak located at 1.69 eV for the complex 10GR–C${}_{70}$ in the lower panel (a). For increasing fields, the fullerene levels are split and new optical transitions are allowed leading to two resonances in the spectrum. It can be noticed that for light longitudinally polarized, the spectra are enriched with many new transitions coming from nanoribbon–fullerene hybridizing states. This is clear for the structure 10GR–C${}_{60}$ where for photon energies larger than 2 eV the breaking of the degeneracy of the fullerene levels by the field admits new transitions between hybridizing states. In Figure 8, we show the band structure of the complex 10GR–C${}_{70}$ in the fat-band representation, which allows a better visualization of the electronic contribution of the carbon atoms from the nanoribbon and those from the atoms of the C${}_{70}$. Columns (a) and (b) show the case of zero electric field and columns (c) and (d) show the case of an applied electric field of 0.5 V/Å. The contribution to the band structure of the 10GR carbon atoms is displayed in (a) and (c) and the contribution of the C${}_{70}$ carbon atoms is displayed in (b) and (d). The biggest circle means a major contribution of the corresponding atoms to a specific point in the band structure.

### 2.3. Graphene-Based Hybrid Structures as Components of Solar Cells

Theoretical studies on the possible use of GNRs as low band gap donor materials for solar cells had been reported by S. Osella et al. [18]. In their paper, they proposed finite GNRs of different shapes and widths which could meet the required electronic and optical properties to be a good candidate for C${}_{60}$-based solar cells. The former results on the optoelectronic properties of graphene-based hybrid structures give support to the idea of using them as possible donor–acceptor systems for organic photovoltaic cells. To perform a qualitative study, we have included an analysis on the type of energy band alignment between the nGRs (donor) and the fullerenes (acceptor) studied.

**Figure 9:**
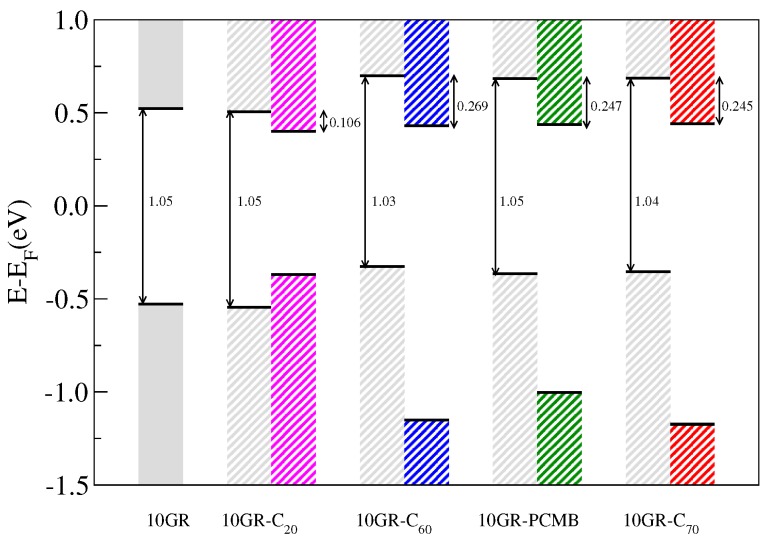
Schematic representation of the band gap of a 10GR nanoribbon and the band alignment of the different 10GR–fullerene hybrids.

**Figure 10:**
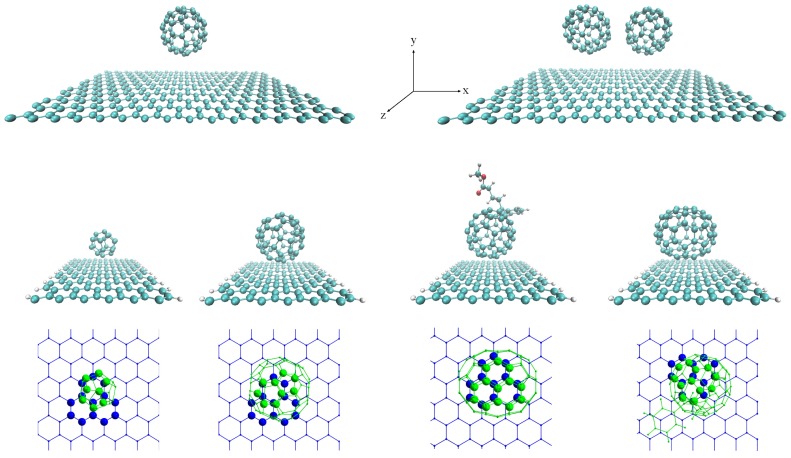
Schematic view of different complexes of fullerenes physisorbed onto a graphene monolayer and armchair graphene nanoribbons. On top SLG–C${}_{60}$ and SLG–2C${}_{60}$ and at the middle from left to right: nGR–C${}_{20}$, nGR–C${}_{60}$, nGR–PCMB and nGR–C${}_{70}$. At the bottom we show the coordination modes for each system.

Figure 9 shows a schematic representation of the alignment of the highest valence band energy (HVBE) and lowest conduction band energy (LCBE) in the Γ point associated with the 10GR and the HOMO and LUMO molecular levels of the different fullerenes considered in the complex 10GR–fullerene. In the scheme, we observe changes in the position of the HVBE and LCBE with respect to the Fermi level ($E_{F}=0$ in this case) when the fullerenes are physisorbed; in particular, for fullerenes C${}_{60}$, PCMB and C${}_{70}$ the HVBE and LCBE are shifted about 0.22 eV with respect to the Fermi level. However, in the case of the smallest fullerene C${}_{20}$, the position of the LCBE and HVBE energies remains almost invariant, and this is because these shifts are caused by the redistribution of charge originated by the van der Waals interaction between the components of the complex, which grows with the size of the fullerene. On the other hand, it can be observed in Table 1 that the difference between the LCBE and the HVBE energies virtually does not change with the presence of the fullerenes and that the difference in energy between the HOMO and LUMO states associated with each fullerene only shows minor changes of at most 0.04 eV. For the systems 10GR–C${}_{60}$/–PCMB/–C${}_{70}$, we observe that the bands display a type-II alignment, which is an ideal characteristic for photovoltaic device operations [2]. Another important aspect that emerges from the scheme shown in Figure 10 is the values of the band offset of the conduction bands. These are fundamental parameters in the development of heterostructured devices [19], where few meV values are needed to favor the electron transfer from the photoexcited donor to the ground-state acceptor [18]. Finally, the band offset and the donor band gap could be employed to determine the maximum quantum conversion efficiency of an excitonic solar cell [20]. For our systems, we get a maximum conduction band offset ($\Delta E_{c}$) of 0.27 eV corresponding to the 10GR–C${}_{60}$ system and a minimum $\Delta E_{c}$ of 0.11 eV corresponding to the 10GR–C${}_{20}$ system, which is not a system with type-II band alignment. Despite the fact that our results do not include many body corrections such as the GW or the Bethe–Salpeter equation (BSE), which are necessary to calculate the correct position of the conduction bands and a best value to the optical band gap, they are a first qualitative approximation for the analysis of possible candidates to be used as excitonic solar cells. This level of theoretical calculation was employed previously to study other heterojunctions for photovoltaic devices [20].

## 3. Materials and Methods

In this work, two different types of configurations are analyzed: C${}_{60}$ fullerenes physisorbed on a graphene monolayer and armchair graphene nanoribbons functionalized with different fullerene molecules C${}_{20}$, C${}_{60}$, C${}_{70}$ and C${}_{60}$ derivative, PCMB. Figure 10 shows a schematic view of the different complexes. Additionally, in this figure, we show the coordination mode (CM) of each complex. In general, the molecules on a complex could be associated in different ways, however a previous study shows that for C${}_{6}0$ physisorbed on small graphene flake, molecules have five possible coordination modes [21]. Of these CMs, the least energy is obtained when a carbon–carbon bond is formed between two hexagons of C${}_{60}$ with central phenyi ring of coronene. For our system at the bottom of Figure 10, we show the approximate coordination mode of the relaxed complex; here, we observe that for all systems, the fullerenes tend to make bonds directly with a carbon atom in the rings of the SLG or nGRs.

Our DFT calculations were carried out by using the SIESTA ab initio package, reference [22] which employs norm-conserving pseudopotentials and localized atomic orbitals as the basis set (double-*ζ*, single polarized in the present work). The fullerene physisorption on SLG and GR is assessed by the van der Waals density functional as proposed by Dion et al. [23] (equivalent to vdW-DF1), which has led to a good agreement between experimental and theoretical results for benzene and C${}_{60}$ physisorbed on graphene [14]. This functional also shows good results for other small molecules physisorbed on graphene [24]. The supercell approach with periodic boundary conditions along the *x* and *y* directions for a SLG and the *z* direction for GRs was employed. For the SLG, we used an 16×5×1 orthogonal supercell, and for the GRs we employed a 1×1×5 supercell. These supercells, together with a vacuum of 15 Å, ensure non-interaction between fullerene images. The systems were fully relaxed by the conjugate gradient minimization until the forces on the atoms were less than 0.05 eV/Å. To guarantee the convergence of the total energy, we have employed a Monkhorst–Pack grid of 3×3×1 for pristine SLG and for SLG–fullerene complexes and of 1×1×5 for pristine GRs and nGRs–fullerene complexes. The fullerene binding energies were calculated by the energy difference between adsorbed and separated constituents, considering corrections due to the basis set superposition error, which is: $E_{b}=E_{AB}^{AB}-E_{A}^{AB}-E_{B}^{AB}.$ Here, the superindex AB represents the basis set of the complex, the subindex AB represents the complex, the subindex *A* represents a SLG or a nGR and the subindex *B* represents a fullerene.

The optical absorption spectrum was obtained through the dipolar approximation implemented in the SIESTA code. For each one of the systems, we have employed a Gaussian broadening of 0.06 eV and a k-points grid of 21×35×1 for pristine SLG and for SLG–fullerenes complexes and of 1×1×51 for pristine GRs and fullerene–GR complexes. These k-points meshes ensure negligible variations in the calculations of the total density of states and the imaginary part of the dielectric function.

## 4. Conclusions and Summary

In this work, we have presented theoretical calculations of the electronic and optical properties of graphene–fullerene nanohybrids in the framework of the density functional theory using the SIESTA package including the van der Waals dispersive interactions. We have studied two different hybridized complexes: C${}_{60}$ fullerenes physisorbed on a graphene monolayer and graphene nanoribbons functionalized with fullerene molecules such as C${}_{20}$, C${}_{60}$, C${}_{70}$ and C${}_{60}$ derivative PCMB. Our results show that the adsorption of the C${}_{60}$ fullerenes on the graphene layer does not alter its low energy states, but its optical response is strongly modified showing new absorption peaks in the visible energy region. The linear absorption spectra for composites formed by fullerenes adsorbed on armchair graphene nanoribbons (GR) cover a broad energy region of the optical spectrum, for longitudinally and transversely polarized photons. A remarkable result obtained from our calculations is that the longitudinally polarized electro-absorption spectrum is enriched with many new transitions coming from nanoribbon–fullerene hybridized states. The knowledge of the band structure of the different complexes provided us the possibility to analyze and to detect those optical resonances derived from transitions between levels of an energy-band of the nanoribbon to energy levels associated to a fullerene.

## Figures and Tables

**Table 1:** Geometrical and electronic parameters of bare fullerenes, pristine graphene and nGRs, and the complexes fullerene–graphene and fullerene–nGR. *d* is the minimal distance between the components of the complexes, $E_{b}$ is the binding energy, $E_{g}$ the band gap of the nGRs and H-L the HOMO and LUMO of the fullerenes.

System	*d*(Å)	$E_{b}$(eV)	$E_{g}$(eV)	H-L(eV)
8GR–C${}_{20}$	2.729	0.44	0.21	0.78
8GR–C${}_{60}$	2.265	0.54	0.20	1.55
8GR–PCMB	2.916	0.72	0.19	1.44
8GR–C${}_{70}$	2.677	0.71	0.21	1.61
10GR–C${}_{20}$	2.700	0.48	1.05	0.93
10GR–C${}_{60}$	2.060	0.45	1.03	1.58
10GR–PCMB	2.600	0.69	1.05	1.44
10GR–C${}_{70}$	2.404	0.76	1.05	1.61
12GR–C${}_{20}$	2.863	0.49	0.62	0.77
12GR–C${}_{60}$	2.772	0.53	0.60	1.59
12GR–PCMB	2.985	0.67	0.63	1.42
12GR–C${}_{70}$	3.001	0.69	0.66	1.57
SLG–C${}_{60}$	2.903	0.74	0.00	1.57
